# B cells in the balance: Offsetting self-reactivity avoidance with protection against foreign

**DOI:** 10.3389/fimmu.2022.951385

**Published:** 2022-07-25

**Authors:** Clara Young, Angelica W. Y. Lau, Deborah L. Burnett

**Affiliations:** ^1^ Immunology Division, Garvan Institute of Medical Research, Darlinghurst, NSW, Australia; ^2^ St Vincent’s Clinical School, Faculty of Medicine, University of New South Wales, Darlinghurst, NSW, Australia

**Keywords:** somatic hypermutation, autoantibody redemption, B cell tolerance, germinal center, affinity maturation, autoreactivity, anergy

## Abstract

Antibodies are theoretically limitless in their diversity and specificity to foreign antigens; however they are constrained by the need to avoid binding to self. Germinal centers (GC) allow diversification and maturation of the antibody response towards the foreign antigen. While self-tolerance mechanisms controlling self-reactivity during B cell maturation are well recognized, the mechanisms by which GCs balance self-tolerance and foreign binding especially in the face of cross-reactivity between self and foreign, remain much less well defined. In this review we explore the extent to which GC self-tolerance restricts affinity maturation. We present studies suggesting that the outcome is situationally dependent, affected by affinity and avidity to self-antigen, and the extent to which self-binding and foreign-binding are interdependent. While auto-reactive GC B cells can mutate away from self while maturing towards the foreign antigen, if no mutational trajectories allow for self-reactive redemption, self-tolerance prevails and GC responses to the foreign pathogen are restricted, except when self-tolerance checkpoints are relaxed. Finally, we consider whether polyreactivity is subject to the same level of restriction in GC responses, especially if polyreactivity is linked to an increase in foreign protection, as occurs in certain broadly neutralizing antibodies. Overall, the outcomes for GC B cells that bind self-antigen can range from redemption, transient relaxation in self-tolerance or restriction of the antibody response to the foreign pathogen.

## Introduction

In a world of virtually limitless number of antigenic possibilities, it is unsurprising that a significant overlap of antigenic composition and structural epitopes can be found on those presented on pathogens and those expressed on self-tissues. Whilst B cells have evolved with the ability to undergo B cell receptor (BCR) rearrangements during early development and in the periphery to combat the inevitable antigenic similarities and diversity, the competing interests of balancing self-tolerance while maintaining foreign protection are undeniably complex. Despite this inherent overlap in the majority of instances B cells are able to generate highly specific foreign binding antibodies while avoiding self-reactivity. The exact mechanisms by which these B cells affinity mature and to what extent this avoidance of self-binding is at the expense of reduced binding, is not yet fully elucidated. Complex experimental models and repertoire sequencing datasets in recent times highlight that a variety of factors may be at play, depending on the complexity of foreign antigen and its similarity to self, genetic factors and antibody stability. In some scenarios, in the process of avoiding self-reactivity, B cells may restrict their response to foreign pathogens. However, in other situations there appears to be demonstratable evidence of self-reactivity forming no barrier, or even providing a selective advantage to B cells maturing to foreign antigens near-identical to self. In this review we have sought to explore the mechanisms of B cell tolerance that might affect the response to foreign pathogens and have highlighted some of the complexity and paradoxical evidence provided by experimental models defining the role of B cell response to pathogens mimicking self.

## Central and peripheral self-tolerance shapes the B cell repertoire

Astonishing diversity within the pre-immune antibody repertoire is achieved by the stochastic recombination of germline immunoglobulin genes; generating an estimated 10^12^ unique specificities during early B cell development (1–5). The price of such an efficient and random process, however, inevitably results in primary BCR rearrangements with significant reactivity against a variety of soluble, cellular, or structural self-antigens (6). While self-reactive early B cells form a majority of the pre-immune antibody repertoire, nature has evolved elegant counter-selection mechanisms during early B cell development.

Developing B cells in the bone marrow carrying a BCR with high affinity or high avidity binding to surface self-antigens, are either clonally deleted or undergo the process of receptor editing (7–16). Alternatively, potent ligation by self-antigens through the immature self-reactive BCR reactivates the recombinase-activating genes to initiate immunoglobulin V(D)J rearrangements in an attempt to reduce self-reactivity followed by further rounds of clonal selection (17, 18); as a result, the revised self-reactive B cells escape clonal deletion and incidentally contribute to the increasing diversity of the primary B cell repertoire. These central tolerance processes of clonal deletion and receptor editing inhibit strongly self-reactive B cells from escaping into the peripheral circulation.

However, despite multiple brakes cohesively censoring self-reactive B cell clones during B cell development in the bone marrow, between 6-30% of B cells with reactivity to self-antigens enter the periphery (7, 19, 20). For example, the germline IGHV4-34 heavy chain immunoglobulin which confers potentially pathogenic autoantibody reactivity by binding N-linked N-acetyllactosamine expressed by the l/i blood group of self-glycoproteins present on red blood cells and B cells, in fact encodes 5% of the B cell repertoire in a healthy individual (21–23). Instead of being deleted, these self-reactive B cells are maintained in a state of reduced responsiveness or “anergy” triggered by continuous BCR signaling (7, 9, 24–32). The discovery of anergy raised the following question – why would self-reactive B cells, with the potential to produce harmful autoantibodies, be maintained in the circulation? This question is perhaps heightened by the fact that anergy is an active process, requiring ongoing BCR engagement and signaling, and thereby is inherently not infallible (33, 34).

The answer likely lies in the hypothesis that anergy serves a dual purpose, and that anergy is used as a compromise by the immune system to firstly avoid generating “holes” within the B cell repertoire which could be exploited by pathogens and secondly, to retain these cells within the repertoire for defense against foreign antigens that structurally resemble self (35–37). If self-reactive anergic B cells are actively maintained in circulation, it suggests that anergic self-reactive B cells must be available to partake in B cell responses against foreign antigens, at least in some circumstances.

## The germinal center response to foreign antigens

During immune responses to foreign pathogens, activated B cells are recruited to germinal centers (GCs) to facilitate affinity maturation towards the foreign antigen. In order to achieve high affinity foreign binding, GC B cells undergo highly competitive rounds of somatic hypermutation (SHM) of their immunoglobulin variable region genes where they engage with foreign antigens presented on follicular dendritic cells and stimuli delivered by T follicular helper cells (Tfh) (38–43).

The mechanisms driving selection of GC B cell clones that bind the foreign antigen with high affinity have been described in detail in several recent reviews (42, 44–47). In brief, the current prevailing mechanism for selection of B cells with high affinity for the foreign antigen is thought to be driven by competition for limited Tfh cell help. GC B cells that carry a BCR which acquired higher affinity for foreign antigen *via* random SHM of their BCR become the most efficient at capturing and presenting the foreign antigen to cognate Tfh cells *via* MHC class II, and as a result, these GC B cells receive a strong survival signal (39, 48). The GC B cells with low affinity for the foreign antigen either submit to apoptosis i.e., “death by neglect” or undergo lower rates of proliferation and eventually become out-competed over time, because they are not able to receive sufficient Tfh-derived stimuli (49, 50). Recent studies have demonstrated that GC positive selection is also driven by isotype class-switching to IgG, which occurs independent of BCR affinity (51, 52). Overall, the final result is that high affinity class-switched GC B cells are selected for differentiation into antibody-producing plasma cells (41, 53–58). In this way, the GC response results in an affinity matured class-switched antibody response against foreign antigens.

## Avoidance of self-reactivity in the germinal center

While the mechanisms governing GC selection against foreign antigens are well studied, the mechanisms which govern avoidance of self-reactivity in the GC, remain largely undefined. During a GC response, there are two major pathways leading to self-reactivity. The first pathway is the *de novo* generation of self-reactivity *via* SHM and the second is through the recruitment and expansion of cross-reactive naïve self-reactive precursor B cells during an anti-foreign response.

In the scenario where a self-reactive B cell is generated *de novo* in the GC, the acquisition of self-reactivity through SHM can sometimes decrease in foreign antigen binding, triggering their “negative selection” due to reduced competitiveness during affinity-based selection. In instances where de novo acquisition of GC self-reactivity also concurrently increases foreign binding, experimental models have shown self-reactive GC B cells are counter selected (59). Thus, the requirement to maintain foreign-binding is critical during decision-making in GC self-tolerance mechanisms. However, what remains largely unknown, is the mechanisms driving “negative selection” of the GC B cells that increase specificity for foreign but also cross react with self-antigens.

Despite our lack of understanding towards the mechanism, the absence of sustained autoantibody production in most individuals clearly indicates that self-tolerance mechanisms do shape the GC-derived antibody repertoire. This extraordinary capacity of B cells to achieve foreign binding while avoiding self-reactivity of cross-reactive antigens is well evidenced in the antibody response to *Campylobacter jejuni*. The vast majority of individuals are able to generate antibodies that effectively bind foreign lipooligosaccharide, while effectively avoiding specificity for near identical self-gangliosides that could result in autoimmune Guillian-Barré syndrome (60–62). However the mechanism driving this exquisite self/foreign discrimination of this antigen remains undefined.

## Balancing tolerance and pathogen response through autoantibody redemption

**Figure 1:**
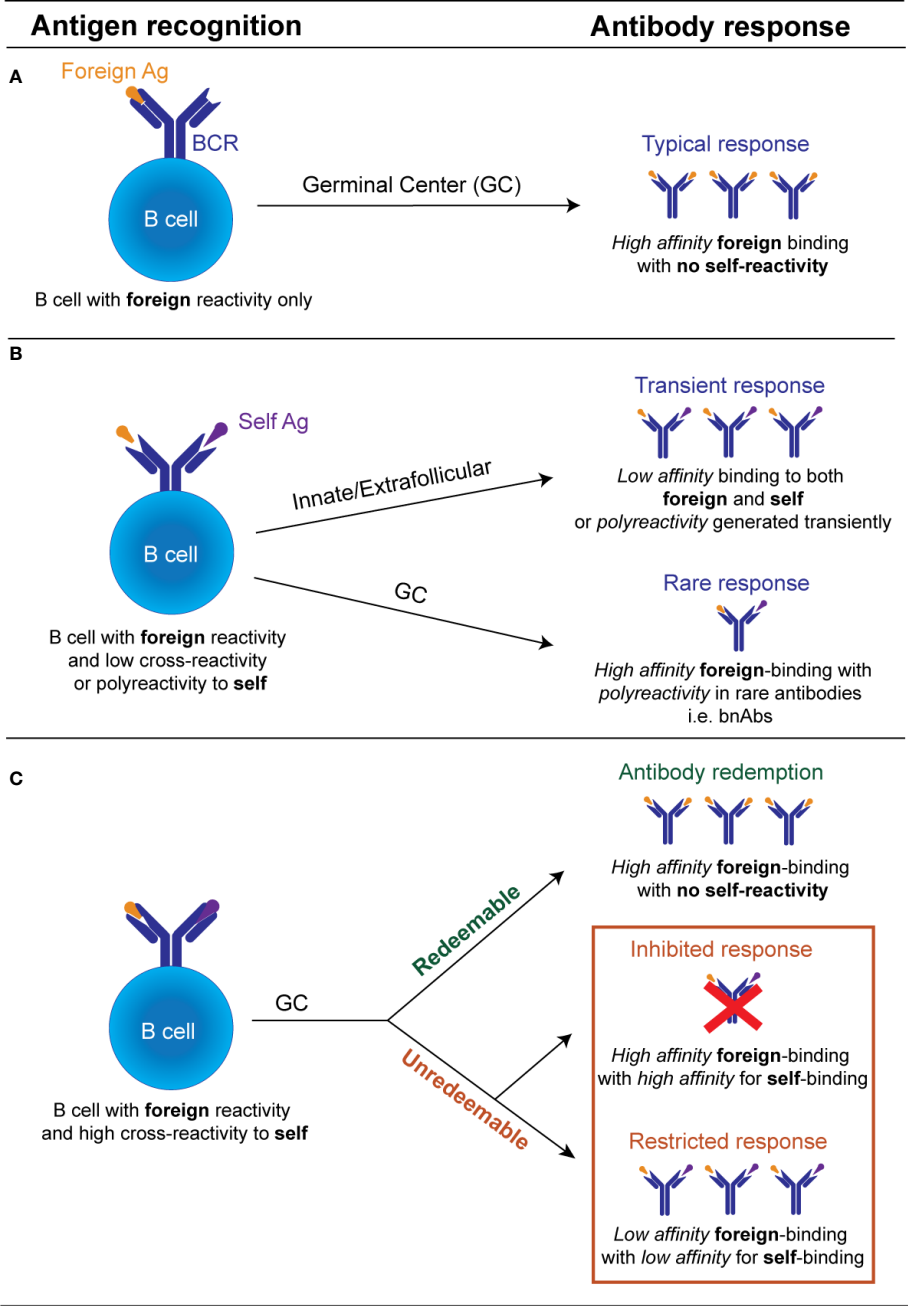
Schematic illustration of the possible fates of an individual antibody receptor encountering self *versus* foreign antigen. **(A)**. If a B cell possesses an antibody receptor which binds strongly to the foreign antigen, but exhibits minimal binding of the self-antigen and minimal polyreactivity, there is no restriction of entry into the GC. Within the GC, B cells undergo affinity maturation of its BCR and affinity-based selection. If this B cell acquires mutations that increase self-reactivity *de novo* it undergoes negative selection by self-tolerance mechanisms. GC B cell with improved anti-foreign binding without detectable increased self-reactivity undergo positive selection to produce high affinity anti-foreign antibodies. **(B)**. If a B cell binds to a foreign antigen but possesses low cross-reactivity or polyreactivity to self-antigens it may still be recruited transiently into the early extrafollicular plasmablast response without entering the GC, releasing unmutated germline polyreactive antibodies. *De novo* generated GC B cells bearing low self-reactivity or polyreactivity may also leave the GC as terminally differentiated antibody-producing plasma cells, likely in situations where the foreign antigen binding is inherently linked to their polyreactivity properties e.g. broadly neutralizing antibodies. **(C)**. B cell with high cross-reactivity to both self and foreign antigens can enter the GC response but largely by-passes recruitment into the early extrafollicular response. Within the GC, this B cell acquires mutations that reduce its autoreactivity. If mutational trajectories are found which decrease self-binding while concurrently maintaining or enhancing foreign binding, these will undergo positive selection and eventually outcompete mutational trajectories which concurrently decrease self and foreign binding. If no mutational trajectories are possible which reduce autoreactivity while concurrently maintaining foreign binding, B cells with high self-binding will be deleted or outcompeted. Only B cells with diminished binding to both the self and the foreign antigen will be positively selected. The processes that facilitate the selection of GC B cells with reduced foreign and self-binding, are currently undefined.

It is now recognized that in certain situations this avoidance of self-reactivity can be facilitated through the process known as “autoantibody redemption” (**Figure 1**). Autoantibody redemption refers to the phenomenon whereby anergic self-reactive B cells enter the GC response and selectively acquire mutations which decrease self-binding. This process was originally described using models which traced the antibody mutation trajectory of HyHEL10 B cells in mice expressing variants of self-antigens Hen Egg Lysozyme (HEL) as model self-antigens immunized with variants of HEL with varying degrees of self-reactivity (36, 63, 64). “Redeemed” self-reactive B cells have since also been found as a component of the antibody response to vaccinia virus and the rhesus D alloantigen (RhD) responses (65), and the response to malaria, whereby a number of B cell clones have shown evidence of a large insertion of LAIR1, which abolishes binding to self-collagen (66, 67).

Interestingly, in some cases, avoidance of self-reactivity by autoantibody redemption during GC responses may actually result in improved antibody responses to the foreign pathogen (**Figure 1**). In the responses to malaria and RhD alloantigen described above, the pre-immune antibodies showed limited binding to the foreign antigen but were significantly increased in the final post immune antibody repertoire (65–67). The HyHEL10 mouse models have shed further light on this phenomenon, revealing that in certain instances the presence of a self-antigen forced the self-reactive B cells to explore different mutational trajectories to those explored by their non-self-reactive counterparts. The alternative mutational pathways undertaken by the self-reactive precursors were beneficial and resulted in higher affinity outcomes than in the absence of self-antigen (36, 37, 64). This indicates that counterintuitively, in some cases, self-reactive precursors may instead generate antibody responses to the foreign antigen that are improved, rather than restricted. This observation suggests targeting certain anergic self-reactive B cells in the periphery may be the key in designing vaccine strategies to generate broadly neutralizing antibodies for self-mimicking pathogens.

## Evidence that avoidance self-reactivity restricts the foreign binding response

Despite the above evidence that in some situations avoidance of self-reactivity can enhance the foreign binding response, an equivalent body of evidence exists that for certain antigens self-reactivity cannot be removed or “redeemed,” and in these instances, on most occasions self-tolerance prevails over foreign protection (**Figure 1**). Evidence from the HyHEL10 model antigen system has shown that when there is no pathway to avoid of self-reactivity while increasing foreign binding, the B cell clones mutate away from both self and foreign binding, and thus are referred to as “unredeemable” antibodies (63, 64).

The phenomenon of “unredeemable” antibodies has also been recognized in the human antibody repertoire. When IGHV4-34 B cells paired with IGLV3 light chains, acquire mutations that remove binding to self-poly-N-acetyl-lactosamine, these mutations also simultaneously removed binding to foreign RhD (65, 68). The inability to redeem self-reactivity for foreign binding, is also likely a key factor limiting the production of broadly neutralizing antibodies e.g. broadly neutralizing antibodies to HIV. Despite an extraordinary SHM mutational profile, some broadly neutralizing antibodies to HIV are unable to remove self-binding to N-linked glycans without simultaneously reducing foreign binding (69, 70).

The predominance of self-tolerance over foreign binding is also highlighted by experiments using the HyHEL10 mouse model system. Even when the B cells do not express a pre-existing BCR that binds to self-antigen, they fail to acquire mutations which increase both foreign reactivity and self-binding (59).

In addition to avoidance of self-reactivity, affinity maturation against foreign also appears to be restrained by host mechanisms to avoid antibodies with reduced solubility, at the risk of developing cryoglobulinemia (19). A specific example has been recognized with the CNTO607 monoclonal antibody, which acquires CDR3 mutations which increase solubility, but at the extent of decreasing foreign binding (71, 72). GC B cells that acquired somatic mutations which damage structural immunoglobulin integrity are eliminated *via* apoptosis (45, 49, 73–75). Hence, antibody responses to foreign pathogens may also be restricted by an attempt to eliminate antibodies with solubility issues which could become pathogenic. However, unlike self-reactivity, it is unlikely that mechanisms that prevent antibody solubility would ever be exploited for vaccine design as the risk of pathogenicity is too high.

## Facilitating foreign binding at the expense of relaxation of self-tolerance

Interestingly several studies have shown for certain antigens, anergic self-reactive B cells can be reawakened when immune checkpoints are dysregulated to disrupt self-tolerance, consequently allowing recruitment in the GC and selection of high-affinity and broadly neutralizing anti-foreign B cell clones. Case studies have shown patients with systemic lupus erythematous (SLE) and certain autoimmune mouse models, including the MRL and LPR mice which lack FAS, are more readily capable of generating broadly neutralizing antibodies to HIV (76–79). Similarly, repertoire sequencing data from SLE patients indicates these patients may have advantages in generating broadly neutralizing antibodies to influenza following seasonal influenza vaccination (80). Immunization with HIV antigen in combination with antibodies inhibiting tolerance checkpoints e.g. CTLA-4 blockade or OX40 agonists, increased the GC response and enhanced production of neutralizing antibodies to HIV (81). Recruitment of anergic B cells for broadly neutralizing antibody generation has also shown to occur in non-autoimmune settings using antigen multimerization (82), additional adjuvants or mitogenic signals (70, 83–85) and T cell stimulation (86, 87). It has also been suggested that immune tolerance may become relaxed following severe COVID19, demonstrated by an increase in circulating B cells with autoreactive antibodies specificities in convalescent patients and evidence that anergic B cells may display a heightened state of activation (88). Although much of this immune activation likely results from combinatorial factors including inflammatory cytokines, specific evaluation of self-reactive IGHV4-34 B cells suggests antigen cross-reactivity may contribute to the expansion of autoreactive B cells in COVID19 (89).

## Permissibility of GC responses to polyreactivity

In addition to playing a role in the innate-like early response to pathogens, polyreactivity also appears to be a feature of some GC-derived antibodies (**Figure 1**). Polyreactive antibodies have the ability to bind several different self-antigens with low affinity. This is in contrast to self-reactive autoantibodies that only bind one self-antigen with moderate to high affinity. Since polyreactivity is conflated with self-reactivity in many studies, it can be difficult to interpret and distinguish the outcome of a polyreactive GC B cell compared to a self-reactive GC B cell. Despite this, there is some evidence to suggest that unlike self-reactivity, GC responses may be permissive to polyreactivity, if polyreactivity is exclusively linked to an increase in foreign antigen.

The immune system appears to tolerate some level of polyreactivity. B cells expressing low-affinity polyreactive antibodies e.g. “natural innate-like B1” B cells, splenic marginal zone B cells and extrafollicular, short-lived plasmablasts, are considered important for the first line of defense against pathogens producing a transient wave of polyreactive antibodies (6, 90, 91). The IGHV1-69 immunoglobulin gene is encoded by germline polyreactivity thanks to a hydrophobic motif in CDR2 capable of binding hydrophobic grooves frequently found within antigens of unrelated foreign pathogens e.g. influenza, hepatitis C virus (HCV), *Staphylococcus aureus* and HIV (92–96). As such germline IGHV1-69 has been proposed to form part of the immediate “SOS” antibody response driving control of certain infections e.g. seasonal influenza, before affinity-matured antibody responses are able provide high affinity protection (97–99).

While polyreactivity is tolerated during short-lived antibody responses, the extent to which polyreactivity is tolerated in GC-dependent long-term antibody responses, is less clear. There is some debate regarding the contribution of the GC response to polyreactivity. Polyreactivity has been reported to occur in up to 25% of IgG^+^ memory B cells yet only ~6% of naïve B cells (100), which indicates the GC response generates polyreactivity. However, the reverse also been reported. In one study, only ~1-2% of IgG^+^ memory B cells and plasma cells demonstrated polyreactivity, representing a decrease of roughly 2-4 fold compared to the naïve B cell compartment (19). These conflicting reports may be explained by differences in experimental assays measuring polyreactivity. Alternatively, they may be explained by the observation that early IgG^+^ memory B cells (typically GC-independent memory B cells) were found to be highly polyreactive (~33%), while late IgG^+^ memory B cells were devoid of polyreactivity (101). Thus, despite the contradictory reports, it appears that overall, the long-term GC-dependent antibody response is not characterized by polyreactivity. Whether this polyreactivity is actively removed during the GC response *via* autoantibody redemption similar to self-reactive GC B cells, or indirectly as a consequence of affinity maturation, remains unknown.

While GC responses are generally associated with reduced polyreactivity, there is evidence to suggest polyreactivity may be permitted in GC responses when polyreactivity is linked to foreign binding affinity through “unredeemable” mutations. Polyreactivity is a recurring feature of broadly neutralizing antibodies, especially those directed to HIV-1 and the stalk region of influenza haemagglutinin (102–105). Roughly 95% of anti-haemagglutinin antibodies targeting a broadly neutralizing epitope at the haemagglutinin stalk region, were found to be polyreactive (105). By contrast, anti-influenza antibodies derived from adults either infected with or vaccinated against seasonal influenza, did not demonstrate significant polyreactivity (105). Interestingly, almost a fifth of the broadly neutralizing anti-haemagglutinin stalk antibodies with polyreactivity were encoded by IGHV1-69 versus <3% of non-broadly neutralizing antibodies (105). Most studies indicate the polyreactivity associated with broadly neutralizing antibodies is germline-encoded, meaning polyreactive precursors are recruited, rather than generated by GC responses. Roughly 70% of polyreactive anti-gp140 HIV-1 antibodies maintained their polyreactivity following reversion to germline (102). Similarly, polyreactive anti-haemagglutinin antibodies reverted to germline demonstrated a reduction in affinity for foreign haemagglutinin binding, yet levels of polyreactivity remained unchanged (105). In line with this, SHM is generally associated with a reduction in polyreactivity (19). The high prevalence of polyreactivity amongst broadly neutralizing antibodies demonstrates the GC response can be permissive to polyreactivity, if associated with an increase towards foreign binding.

Thus, this indicates polyreactive GC responses may not be restricted by “unredeemable” mutations like self-reactive GC responses. In support of this, polyreactive antibodies have been shown to demonstrate heteroligation to self and foreign antigen i.e. simultaneous binding to high density self-antigen and low-density foreign antigen (102). One could speculate that polyreactive GC B cells positively selected due to their increased competitiveness for foreign antigen binding, fail to trigger the threshold for self-tolerance that prevails for “unredeemable” self-reactive GC B cells, because affinities for self-antigen are too low. Future studies are needed to elucidate the fate of polyreactive B cells during the GC response including “unredeemable” polyreactive GC B cells, particularly since some of the coveted broadly neutralizing antibodies to influenza and HIV are associated with polyreactivity.

## Conclusion

In this review we have sought to explore dualistic and unique challenges faced by B cells combating foreign pathogens that cross-react with self-antigens. We highlight several recent studies in the field that have championed our progress in understanding how GC B cells mitigate antigenic cross-reactivity through the process of autoantibody redemption. However, more studies are required to further address some major outstanding questions in the field, to allow for the adaption of this understanding to benefit vaccine design and pathogen defense.

Autoantibody redemption, at least in experimental mouse model systems, can lead to beneficial and higher affinity outcomes to the foreign antigen. Although it has been shown to occur in both natural infections and vaccination responses it remains currently unknown how frequently these processes occur and whether the process of autoantibody redemption can be harnessed in vaccine design to enhance the broadly neutralizing antibody response. Ongoing research is required to assess the feasibility of recruitment of anergic self-reactive B cell precursors and driving them towards a “redeemable” mutational trajectory *via* a combination of a germline-targeting and sequential immunization, in the setting of pathogens that thus far remain elusive in universal vaccine development.

Another aspect of the field that remains relatively unexplored, is the fate of polyreactive B cells during GC responses (in clear distinction with self-reactive GC B cells). At a very fundamental level, the field still lacks a clear definition on how much polyreactivity is present in a typical GC-dependent antibody repertoire. Additionally, while it appears polyreactivity is reduced following GC responses, it remains unclear whether polyreactivity is removed *via* an active mechanism e.g. autoantibody redemption similar to self-reactive GC B cells, or is a passive consequence of SHM during affinity maturation. Since many broadly neutralizing antibodies are inherently polyreactive, understanding how GC responses deal with polyreactivity is also pertinent for the field of vaccine design.

Finally, one major roadblock to understanding of how GC B cell responses to foreign antigens might be inhibited by self-antigens, lies in our complete lack of understanding regarding the mechanisms controlling GC B cell tolerance in the face of “unredeemable” mutations. Without elucidating these mechanisms, we cannot fully understand how GC B cell responses to foreign antigens are shaped by cross-reactivity to self-antigens. Understanding how B cells deal with foreign pathogens that mimic self is important for many aspects of B cell biology and including the fields of autoimmunity and vaccine design.

## Author contributions

CY, AL, and DB contributed to conception, design, interpretation and writing of the manuscript. All authors contributed to the article and approved the submitted version.

## Funding

This work was funded by NHMRC Investigator Grant 1176351. C Young was supported by a University of New South Wales (UNSW) Scientia Scholarship for postgraduate study.

## Conflict of interest

The authors declare that the research was conducted in the absence of any commercial or financial relationships that could be construed as a potential conflict of interest.

## Publisher’s note

All claims expressed in this article are solely those of the authors and do not necessarily represent those of their affiliated organizations, or those of the publisher, the editors and the reviewers. Any product that may be evaluated in this article, or claim that may be made by its manufacturer, is not guaranteed or endorsed by the publisher.
